# Synergistic Antimicrobial Activity of Supplemented Medical-Grade Honey against *Pseudomonas aeruginosa* Biofilm Formation and Eradication

**DOI:** 10.3390/antibiotics9120866

**Published:** 2020-12-04

**Authors:** Carlos C. F. Pleeging, Tom Coenye, Dimitris Mossialos, Hilde de Rooster, Daniela Chrysostomou, Frank A. D. T. G. Wagener, Niels A. J. Cremers

**Affiliations:** 1 Small Animal Department, Faculty of Veterinary Medicine, Ghent University, Salisburylaan 133, 9820 Ghent, Belgium; ccfpleeging@gmail.com (C.C.F.P.); hilde.derooster@ugent.be (H.d.R.); 2 Department of Dentistry, Orthodontics and Craniofacial Biology, Radboud University Medical Center, Philips van Leydenlaan 25, 6525EX Nijmegen, The Netherlands; frank.wagener@radboudumc.nl; 3 Dierenkliniek Parkstad, Bautscherweg 56, 6418EM Heerlen, The Netherlands; 4 Laboratory of Pharmaceutical Microbiology, Ghent University, Ottergemsesteenweg 460, 9000 Ghent, Belgium; tom.coenye@ugent.be; 5 Microbial Biotechnology-Molecular Bacteriology-Virology Laboratory, Department of Biochemistry and Biotechnology, University of Thessaly, Biopolis-Mezurlo, 41500 Larissa, Greece; mosial@bio.uth.gr; 6 Wound Clinic Health@45, Linksfield Road 45, Dowerglen, Johannesburg 1612, South Africa; danielachrys@hotmail.com; 7 Triticum Exploitatie BV, Sleperweg 44, 6222NK Maastricht, The Netherlands

**Keywords:** medical-grade honey, supplements, vitamin C, vitamin E, antimicrobial, biofilm, *Pseudomonas aeruginosa*, wound

## Abstract

Biofilms hinder wound healing. Medical-grade honey (MGH) is a promising therapy because of its broad-spectrum antimicrobial activity and the lack of risk for resistance. This study investigated the inhibitory and eradicative activity against multidrug-resistant *Pseudomonas aeruginosa* biofilms by different established MGH-based wound care formulations. Six different natural wound care products (Medihoney, Revamil, Mebo, Melladerm, L-Mesitran Ointment, and L-Mesitran Soft) were tested in vitro. Most of them contain MGH only, whereas some were supplemented. L-Mesitran Soft demonstrated the most potent antimicrobial activity (6.08-log inhibition and 3.18-log eradication). Other formulations ranged between 0.89-log and 4.80-log inhibition and 0.65-log and 1.66-log eradication. Therefore, the contribution of different ingredients of L-Mesitran Soft was investigated in more detail. The activity of the same batch of raw MGH (1.38-log inhibition and 2.35-log eradication), vitamins C and E (0.95-log inhibition and 0.94-log eradication), and all ingredients except MGH (1.69-log inhibition and 0.75-log eradication) clearly support a synergistic activity of components within the L-Mesitran Soft formulation. Several presented clinical cases illustrate its clinical antimicrobial efficacy against *Pseudomonas aeruginosa* biofilms. In conclusion, MGH is a potent treatment for *Pseudomonas* biofilms. L-Mesitran Soft has the strongest antimicrobial activity, which is likely due to the synergistic activity mediated by its supplements.

## 1. Introduction

Wounds that fail to show progress in wound healing during four weeks or are not completely healed within eight weeks are generally considered to be chronic [1,2]. In developed countries, treatment costs for chronic wounds are estimated to account for approximately 1–4% of the total healthcare expenditure, thus imposing a major economic burden [3,4]. Moreover, chronic wounds impact the quality of life and affect the physical and psychological well-being of the patients [4]. The number of patients and costs are expected to further grow with the aging population and increased incidence of comorbidities such as diabetes and obesity [2]. Major types of chronic wounds include diabetic ulcers, pressure injuries, and venous leg ulcers [1,5]. Such prolonged, nonhealing wounds are caused by a variety of factors, with bacterial infections and especially biofilm formation being significant contributors [2,3]. Neglected wound infections can lead to systemic infections, sepsis, and multiple organ dysfunction syndrome and become life-threatening [6].

The prevalence of biofilms in chronic wounds is estimated to be approximately 60% [1,7]. Biofilms have been defined as syntrophic clusters of bacterial cells encased in a biopolymer matrix, which show increased resistance to host cellular responses as well as topical (antiseptics) and systemically administered antimicrobials [3,8,9,10]. Biofilm formation is a strategy of microorganisms to successfully adapt and survive in hostile environments, which can increase its resistance to the effects of antimicrobial agents 10–1000 times [10]. The biofilms in chronic wounds often consist of multiple bacterial species, of which *Pseudomonas aeruginosa* and *Staphylococcus aureus* are the most common [11,12]. The prevalence of *Pseudomonas aeruginosa* may be underestimated as these bacteria often reside in the deeper wound regions, and this may subsequently also complicate treatment [13,14]. Chronic wounds that harbored *Pseudomonas aeruginosa* were associated with larger wound sizes and a more severely hindered healing process [11,13]. As a consequence of biofilm formation, *Pseudomonas aeruginosa* has a predilection to develop resistance to antibiotics, and the expression of multiple virulence factors contributes to the frequent ineffectiveness of current therapies [15,16]. *Pseudomonas aeruginosa* PAO1 is the most commonly used *Pseudomonas* strain for research, is one of the top three causes of opportunistic human infections, and highly resistant to antibiotics and disinfectants [17,18]. Novel therapies that combat biofilms and biofilm-related infections, and that carry a reduced risk for the development of antimicrobial resistance are therefore urgently needed [3]. Medical-grade honey (MGH) forms such a promising therapy because of its broad-spectrum antimicrobial activity and the lack of risk for resistance [9].

MGH is strictly controlled and extensively tested honey, guaranteeing its safety and efficacy for medical purposes [19]. MGH exerts both broad-spectrum antimicrobial activity and wound-healing-promoting properties [20,21,22,23]. The main antimicrobial activities of MGH are thought to rely on the low pH, osmotic activity, production of hydrogen peroxide, and the presence of antimicrobial molecules (e.g., phytochemicals such as flavonoids). Since the antimicrobial activity is attributed to multiple mechanisms, no resistance towards honey has been reported so far [24]. The wound-healing properties are multifaceted and mediated by creating a moist wound environment, stimulation of autolytic debridement and angiogenesis, anti-inflammatory and antioxidative activity, enhanced cell migration, proliferation, and re-epithelialization [20,21,22,23]. In a recent systematic review paper about honey in wound care, analyzing 30 randomized controlled studies, it was concluded that honey decreased the time of wound healing and was cost-effective [25]. MGH constantly demonstrates promising results in vitro as well as in the clinic during wound care [2,7,9,26,27]. Interestingly, MGH was also able to efficiently treat an existing MRSA biofilm on a titanium mesh placed in a large incisional ventral hernia and prevented new infections [28]. MGH can also have a prophylactic activity and subsequently will also reduce the development of biofilms [29,30].

The objective of this study was to evaluate the inhibitory and eradicative activity of different MGH-based wound care formulations against multidrug-resistant *Pseudomonas aeruginosa* biofilms formed in an in vitro biofilm wound model [31]. In addition, clinical cases of wounds with presumed *Pseudomonas aeruginosa* infections and biofilms that were effectively treated with MGH will be presented.

## 2. Results

### 2.1. MGH Strongly Inhibits Pseudomonas aeruginosa Biofilm Formation, Which Can Further Be Enhanced by Supplements

To investigate the inhibitory activity of MGH on the formation of multiresistant *Pseudomonas aeruginosa* biofilms, six MGH-based wound care products were tested. The products were placed on the AD directly after *Pseudomonas aeruginosa* cells were inoculated and allowed to grow for 24 h so biofilm formation on the AD could take place.

**Figure 1:**
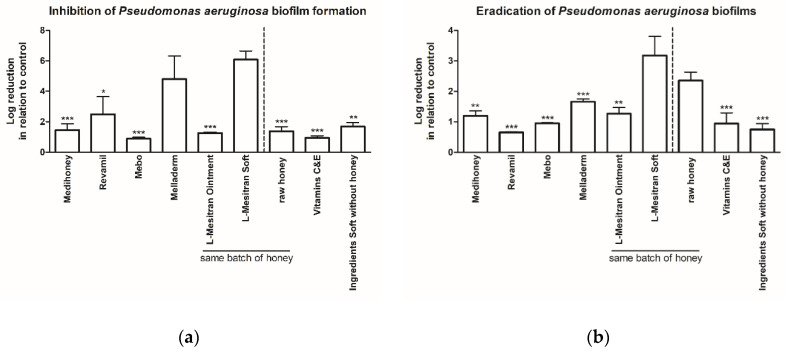
Antibiofilm activity of different natural products on *Pseudomonas aeruginosa* biofilms. Data are presented as mean log reduction ± standard deviation in relation to untreated control. (**a**) Inhibition of *Pseudomonas aeruginosa* biofilm formation. L-Mesitran Soft was significantly more effective (*** *p* < 0.001) compared to all other products, except Melladerm (significances not added in the graph). Significant differences with Melladerm are indicated by * above each column (* *p* < 0.05, ** *p* < 0.01, *** *p* < 0.001). No other significant differences were observed. (**b**) Eradication of *Pseudomonas aeruginosa* biofilms. L-Mesitran Soft was significantly stronger (*** *p* < 0.001) compared to all other groups, except raw honey (significances not shown in the graph). Significant differences with raw honey are indicated by * above each column (** *p* < 0.01, *** *p* < 0.001). Melladerm was significantly higher than Revamil and Ingredients Soft without honey groups (* *p* < 0.05). No other significant differences were observed.

All products demonstrated a significant biofilm inhibitory activity (Figure 1a). The highest inhibitory activity was observed with L-Mesitran Soft (6.08-log reduction compared to untreated control) and Melladerm (4.80-log), both being significantly higher than Medihoney (1.45-log), Revamil (2.49-log), Mebo (0.89-log), and L-Mesitran Ointment (1.26-log). The contribution of the different supplements of L-Mesitran Soft, the most active formulation, was investigated in more detail (Figure 1a). The same batch of MGH as present in L-Mesitran Soft and Ointment (at 40% concentration, similar as in L-Mesitran Soft) showed a 1.38-log reduction, vitamins C and E had a 0.95-log reduction, and for all ingredients without the MGH a 1.69-log reduction was measured. The separate ingredients demonstrated a lower activity on their own than combined in the L-Mesitran Soft formulation, suggesting that they exhibited synergistic activity when combined.

### 2.2. MGH Eradicates Existing Pseudomonas aeruginosa Biofilms, with Increased Efficacy by Supplements

Next, we investigated whether the same wound care products could eradicate preformed *Pseudomonas aeruginosa* biofilms (Figure 1b). Again, L-Mesitran Soft had the most potent antibiofilm activity (3.18-log reduction compared to untreated control), and this was significantly higher (all *p* < 0.001) when compared to Medihoney (1.20-log), Revamil (0.65-log), Mebo (0.95-log), Melladerm (1.66-log), and L-Mesitran Ointment (1.27-log). The contribution of different ingredients of L-Mesitran Soft towards the eradicating effect was further investigated; the same batch of raw MGH showed an eradicating activity of 2.35-log when used alone, which was significantly more effective than all other products. Vitamins C and E and all ingredients except MGH eradicated the biofilms (0.94-log and 0.75-log, respectively). The eradicating activity of each of the ingredients was again lower than that of the L-Mesitran Soft, suggesting synergistic activity.

### 2.3. L-Mesitran Soft Has also the Strongest Antibiofilm Activity against Staphylococcus aureus

To explore whether other bacterial species are also susceptible to MGH-based wound care products, the inhibiting and eradicating activity against *Staphylococcus aureus* (Mu50) was investigated. *Staphylococcus aureus* Mu50 is the first clinical vancomycin-resistant MRSA strain isolated in Japan in 1997 [32]. The inhibitory activity of L-Mesitran Soft was significantly stronger compared to control, Medihoney, Revamil, and Mebo (Figure S1a). L-Mesitran Ointment was significantly stronger than Medihoney (* *p* < 0.05) and Revamil (* *p* < 0.01). No other significant differences were observed. The eradicating activity of L-Mesitran Soft was significantly higher (*p* < 0.05) when compared to the control group (Figure S1b). However, no other significant differences were observed.

### 2.4. Case Reports Illustrating the Antibiofilm Activity in the Clinic

To translate these in vitro findings to clinical relevance, the effect of L-Mesitran Soft on the eradication of established biofilms in wounds predominantly infected with *Pseudomonas aeruginosa* is further demonstrated by several case reports.

#### 2.4.1. Case 1: Infected Diabetic Ulcer

**Figure 2:**
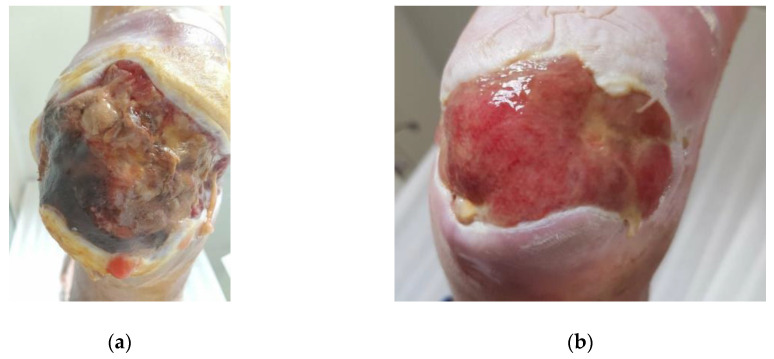
Clinical Case 1 of a *Pseudomonas*-infected diabetic ulcer, located on the heel, treated with L-Mesitran Soft. (**a**) Picture at the start of L-Mesitran Soft treatment on Day 0. (**b**) Wound after one month of treatment on Day 33.

A 44-year-old female patient with type 1 diabetes mellitus presented with a diabetic foot ulcer (DFU), located on the heel, upon referral from her doctor (Figure 2a). Although the patient was obese (BMI 35.7), she stated that she adheres to the diabetic care protocol, i.e., diet, exercise, and medication. HbA1C was within normal limits (6.3) and normal pedal pulses were palpable. The patient was terrified of losing her foot, as the hospital already booked her for amputation. The DFU (Grade 3, Stage B, according to the Texas grading system [33]) was heavily infected with *Pseudomonas aeruginosa*, with typical green staining, its characteristic odor, and copious amount of exudate [34,35]. The DFU had been present for three months, and despite the use of oral antibiotics (Augmentin: amoxicillin and clavulanate), Bactroban dressings (mupirocin), and regular gauzes, the wound was deteriorating and infection persisted.

At the first visit, limited sharp debridement was performed due to severe local neuropathy and ambiguity of structures in the wound bed. Next, a thick layer of L-Mesitran Soft was applied to the wound and covered with a regular secondary absorbent dressing. Because of the excess debris in the wound, the malodor, and excessive levels of exudate, the dressing was changed the following day. The change of the dressing was easy and wound irrigation with saline revealed the presence of patches with solid granulation tissue, and wound care with L-Mesitran Soft was continued. The pressure on the wound was reduced by wearing a special boot. After the next dressing change (Day 3), the malodor was no longer present, and granulation tissue formation further increased, sinuses were revealed, and the calcaneus became exposed. Limited sharp debridement was performed again respecting the structures of the wound. Subsequently, the wound was treated twice a week and continued for one month (Figure 2b, Day 33). The wound size was strongly reduced, with full granulation of the wound bed, and physical and mental quality of life for the patient had improved.

#### 2.4.2. Case 2: Massive Abscess Infected with *Pseudomonas aeruginosa* and *Staphylococcus aureus*

**Figure 3:**
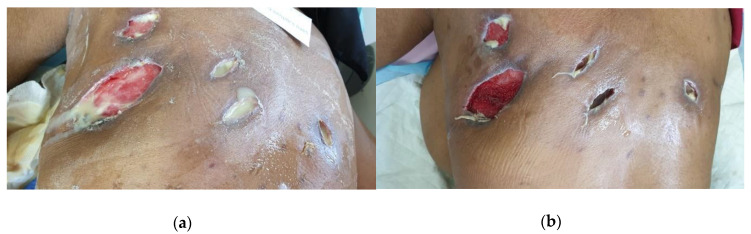
Clinical Case 2 with a massive *Pseudomonas*- and *Staphylococcus*-infected abscess on the back, of which the cavities were filled with L-Mesitran Soft as treatment. (**a**) Picture at the start of L-Mesitran Soft treatment on Day 0. (**b**) Clear wound progression after three dressing changes and one week of treatment.

A 65-year-old female patient with well-controlled type 1 diabetes mellites (HbA1C 6.7) presented to a private clinic with a massive abscess on the back area. The cause of the abscess was unknown, but a private surgeon incised it at multiple sites and drained the content. The patient was supposed to take care of the wound dressings herself, but she and her family were overwhelmed by the copious amount of odorous exudate and needed professional wound care. After three weeks of irrigating the wound with saline and using regular gauzes, the patient presented to the wound clinic for help. At the first visitation (Figure 3a, Day 0), the patient had a low-grade fever, was not feeling well with nausea and dizziness, and had severe pain on the whole back (score of 8 out of 10 on a visual analog scale [36]). She stated that she is depressed, anxious, and stressed, afraid of losing her life. The abscess covers the area of the whole trapezius and part of the latissimus dorsi on the left side of the trunk and upper part of the right trapezius, and wounds are subcutaneously connected. Infection and inflammation were present with a heavy odorous purulent exudate. The tissue was necrotic with the presence of slough and some granulation visible at the base of the wound after irrigation with the edges of the wound rolled and indented. Because of the severity of the infection, a swab was taken to identify the bacterial origin of the infection.

Irrigation of the wound was performed with a saline solution via a tube adapted to a syringe. Subsequently, wound cavities were filled with L-Mesitran Soft and covered with regular nonadhesive, superabsorbent dressings. After two days, odor and exudate levels decreased. Laboratory results confirmed the presence of multiresistant *Staphylococcus aureus* (resistant to clindamycin, erythromycin, fusidic acid, and penicillin) and nonresistant *Pseudomonas aeruginosa*, and because of the uncertain deepness of the abscess, antibiotic therapy with Ciprofloxacin (500 mg BD for 10 days) and Augmentin (1 g BD for 10 days) was initiated to ensure systemic reach. On Day 4, the pain was no longer present (score of 0 out of 10), the general health improved, the viscosity and amount of exudate decreased, and there was no longer malodor. After one week, inflammation further decreased (Figure 3b, Day 7). Wound therapy with L-Mesitran Soft was continued and combined with negative pressure therapy in an attempt to further control the exudate level and completely resolve the infection.

#### 2.4.3. Case 3: Pressure Ulcer

**Figure 4:**
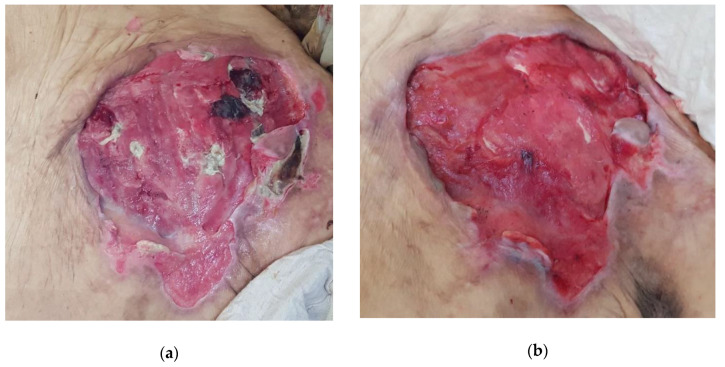
Clinical Case 3 of *Pseudomonas* infected pressure ulcer treated with L-Mesitran Soft. (**a**) Picture at the start of L-Mesitran Soft treatment on Day 0. (**b**) Wound after just one dressing change on Day 3.

An 89-year-old female patient with advanced dementia and poor nutritional state was bedridden and totally dependent on care. She developed a large pressure ulcer of roughly 20 by 15 cm covering a large part of the lumbosacral area after being hospitalized for pneumonia (Figure 4a, Day 0) after which wound care was alas neglected. The patient presented to the wound clinic because of an excessive exuding, odorous, and very painful sacral pressure ulcer. The wound appeared to be infected with *Pseudomonas aeruginosa* based on the green color of copious exudate and its specific odor.

L-Mesitran Soft was applied over the area and covered with a regular non-adhesive, absorbent dressing that was secured with films. After 72 h, the dressing was changed, and the wound already clearly progressed (Figure 4b, Day 3). Necrotic tissue and wound debris almost completely disappeared, and the wound looked more red and vital. In addition, malodor strongly abated. Due to the extreme difficulty in transporting the patient for follow-up and the wound location, a designated carer was trained to perform the dressing changes, with the opportunity to digital assistance. Unfortunately, the patient died shortly after the start of the wound treatment from another episode of pneumonia.

#### 2.4.4. Case 4: Heavily Infected Wound on the Ankle

A 56-year-old male presented to the wound clinic with a large odorous wound covering the anterior and lateral part of the right ankle. The cause of the wound was unclear; however, the patient underwent an ankle surgery about thirty years ago and again last year for treating a fracture of the same ankle. It was not sure if the wound was related to this surgery, but it was at the same location as the old scar. The wound became bigger over several months although the patient was treated by multiple state hospitals with various topical ointments, including Bactroban (mupirocin) and Fucidin, and he received various oral antibiotics on numerous occasions. The patient is a smoker (10 cigarettes per day, and occasionally marihuana).

**Figure 5:**
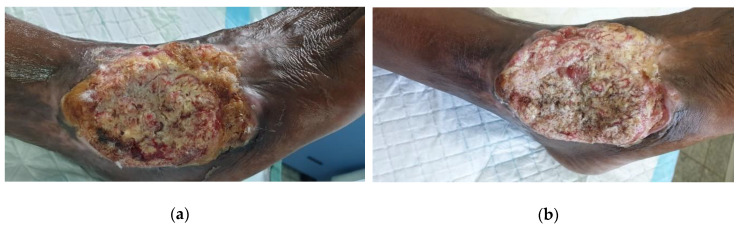
Clinical Case 4 of heavily *Pseudomonas*-infected wound on the ankle treated with L-Mesitran Soft. (**a**) Picture at start of L-Mesitran Soft treatment on Day 0. (**b**) Clear wound progression after 20 days of treatment.

The ulcer was heavily infected with *Pseudomonas aeruginosa*, had the characteristic odor, and produced thick, green, copious amounts of exudate (Figure 5a). The wound was very painful, and the patient used crutches to be able to walk. The wound was suspected to be cancerous due to the tissue structure. This needed to be confirmed with a biopsy; however, the patient refused this. L-Mesitran Soft was applied and covered with an absorbent secondary dressing. The patient was scheduled to come back the following day for a dressing change but did not show up because of low income and he did not want to spend any money on wound care. On Day 3, the wound dressing was changed *pro bono*. The wound was macerated, bled, and the bandages were very dirty due to the excessive amount of exudate and delayed dressing change. On Day 6, the patient came for the second dressing change, and after cleansing the area with saline, the wound odor was decreased and the wound remarkably improved. The same protocol was used for another two weeks without antibiotics. On Day 20, the wound size reduced with the presence of granulation and epithelial tissue, and there was a strong reduction in odor, necrotic tissue, and slough (Figure 5b). Moreover, the pain was reduced, and the patient was able to walk with only one crutch. Against our advice, the patient decided not to come to the clinic anymore and take care of the wound himself.

## 3. Discussion

All tested honey products were highly effective in destroying *Pseudomonas aeruginosa* bacteria and biofilms in the used in vitro biofilm wound model. L-Mesitran Soft demonstrated the highest inhibitory and eradicating activity of *Pseudomonas aeruginosa* biofilms when compared to other natural and MGH-based wound care products (Medihoney, Melladerm, Revamil, Mebo, and L-Mesitran Ointment). The superiority of L-Mesitran Soft regarding its inhibitory and eradicating activity was also observed towards *Staphylococcus aureus* when compared to the same wound care products. The separate ingredients used in L-Mesitran Soft: raw honey, vitamins C and E, and the formulation without honey had antibiofilm activity on its own. However, the combination had synergistic activity, clearly demonstrating that other ingredients can enhance the antimicrobial activity of MGH. Surprisingly, there was a differential effect between L-Mesitran Ointment and L-Mesitran Soft. This was likely due to the different formulations. Possibly, the oils and higher concentration of lanolin in the L-Mesitran Ointment could have inhibited the activity of the vitamins and have interfered. Other options are that the propylene glycol and polyethylene glycol 4000 (PEG 4000) that are only present in the L-Mesitran Soft are the distinguishing factors. To translate these preclinical findings to clinical relevance, case reports were presented that illustrate that, in the clinic, L-Mesitran Soft has a strong activity on *Pseudomonas* biofilms in infected ulcers, and these infections were resolved very fast while simultaneously improving the wound-healing trajectory.

We demonstrated that a combination of vitamins C and E exert a strong antimicrobial activity and reduced *Pseudomonas aeruginosa* biofilms, both on inhibition (88.78%) and eradication (88.59%). However, the raw MGH on its own was even more effective, with a reduction of 95.88% in growth inhibition and 99.56% in the eradication of the biofilms. Interestingly, the combined ingredients used in the L-Mesitran Soft formulation without the MGH had an even stronger inhibitory activity with a 97.94% reduction but a little lower biofilm eradicating activity (82.13%). The strongest effect was observed with the complete L-Mesitran Soft formulation that inhibited 99.9999% of *Pseudomonas aeruginosa* biofilm formation while 99.93% of the established biofilms were eradicated. These data clearly demonstrate that different components of L-Mesitran Soft exert antimicrobial activity. Furthermore, there is a strong synergistic activity when used together in the complete L-Mesitran Soft formulation.

These data are in accordance with previous findings of two independent studies in which the antimicrobial activity of L-Mesitran Soft was compared with the same batch of raw honey used in the formulation [37,38]. Both studies demonstrated that L-Mesitran Soft has a stronger antimicrobial activity than raw honey towards *Staphylococcus pseudintermedius*, *Malassezia pachydermatis*, and *Candida albicans*. These findings support the assumption that the added supplements in the L-Mesitran Soft formulation enhanced its antimicrobial activity [37,38]. In contrast to those studies, we here investigated the contribution of the different supplements in more detail.

In the present study, since we observed the strongest antimicrobial activity using L-Mesitran Soft, we will elaborate on the different ingredients of this formulation and discuss the possible contribution to the antimicrobial and wound-healing activities. L-Mesitran Soft contains 40% medical-grade honey, vitamins C and E, medical-grade hypoallergenic lanolin, PEG 4000, and propylene glycol.

Vitamins C and E are antioxidants that fulfill an important role in protection from oxidative stress and are well-known to further enhance the prohealing effects of MGH [39,40,41,42,43,44,45,46]. Vitamin C is a well-known cofactor in the biosynthesis of collagen and improves angiogenesis and tensile strength in the skin [47,48,49]. Vitamin E protects cells from lipid peroxidation, is anti-inflammatory, and reduces scar formation [43]. Numerous studies have shown that vitamins C and E exert antimicrobial activity against a wide range of microorganisms, including *Staphylococcal* species (including *Staphylococcus aureus*), *Streptococcal* species, *Proteus vulgaris*, *Escherichia coli*, *Bacillus subtilis, Candida albicans*, *Pseudomonas aeruginosa*, and *Klebsiella pneumoniae* [50,51,52,53]. In addition, these vitamins can have synergistic effects with antibiotics and enhance growth inhibition and the lethal effects of antibiotics against microorganisms [40,50,54,55,56,57,58,59,60,61,62,63]. Recent studies emphasize that vitamins C and E can also enhance the antimicrobial activity of honey [24,37,38,50].

Vitamins C and E promote an immunomodulatory effect and increase the antimicrobial sensitivity via diverse mechanisms [63,64]. Low concentrations of vitamin C reduce the synthesis of extracellular polymers that form the bacterial biofilms and destabilize these biofilms, whereas high concentrations of vitamins are also able to kill bacteria [65,66]. Vitamin C is also involved in bacterial metabolism; whereas several bacteria can ferment vitamin C, others cannot result in oxidative stress that will inhibit bacterial growth [64]. Vitamin C likely triggers the intracellular production of reactive oxygen species in bacterial cells [50]. In addition, vitamin C alters the bacterial cell surface to render it increasingly permeable to antibiotics and potentiate the antimicrobial activity [53]. Moreover, the transition of microorganisms from a planktonic to a biofilm-sessile state thrives under oxidative stress, and therefore antioxidants, such as vitamins C and E, could be a potent complementary therapy [67]. A recent study showed that the supplementation of different types of honey with vitamin C resulted in a significant enhancement of the antibacterial activity against *Pseudomonas aeruginosa* and *Escherichia coli* in a dose-dependent manner [50]. In addition, in a multispecies biofilm model with four bacterial species (*Staphylococcus aureus*, *Streptococcus agalactiae*, *Pseudomonas aeruginosa*, and *Enterococcus faecalis*), vitamin C enhanced the antibiofilm effect of honeydew honey against all four isolates within 24 h [50]. Although the antibacterial effects of vitamin C may be both bacterial strain- and concentration-dependent [50], these findings suggest a broad range of antimicrobial activity. Therefore, a combination of honey supplemented with vitamins is a very promising therapy for the treatment of chronically infected wounds and has been increasingly recognized as effective in clinical settings [22,50].

Lanolin is anecdotally claimed to be antimicrobial, but there is little scientific evidence to substantiate this [37]. Next to possible antimicrobial activity, lanolin can contribute to improved wound healing as is demonstrated in experimental wounds and clinical studies on cracked and sore nipples [68,69,70,71,72]. In addition, lanolin is topically applied to treat dry and irritated skin, mainly in the sectors of cosmetics and health care, as it aids in restoring the epidermal barrier and reducing water loss of the skin [73,74,75]. These hydrating properties make it an excellent vehicle for retaining water-soluble pharmaceutical, cosmetic and antimicrobial agents, such as MGH [62,74,75].

PEG 4000, with the number denoting its molecular weight, is a substance widely used as a vehicle or cosolvent in a variety of pharmaceutical, cosmetic, ophthalmic solutions and sustained-released oral pharmaceutical applications [76,77]. In a randomized controlled trial, 84 patients with partial-thickness burns were divided into two equal groups treated either with honey alone or honey fortified with antioxidants (vitamins C and E) and PEG 4000. The burns in the latter group healed faster than the group treated with honey alone (mean of 6.4 days versus 8.3 days) [78]. The same supplements are also used in L-Mesitran Soft, and thus likely will enhance the healing properties of honey as well. PEG is reported to have antimicrobial activity, with PEG 1000 being stronger than PEG 400 [79].

Propylene glycol is often used as a dermatological vehicle for gels and ointments and allows better penetration into the skin and subcutaneous tissues [80]. Moreover, propylene glycol has been shown to possess antimicrobial activity [79,81].

There are multiple mechanisms of action regarding the antimicrobial activity of honey against *Pseudomonas aeruginosa*. Among others, honey induces loss of structural integrity and marked changes in cell shape and surface, possibly by increasing *algD* and decreasing *oprF* gene expression, leading to membrane depolarization and permeabilization and subsequently cell lysis [82,83,84]. Honey reduces the ability to acquire iron by decreasing siderophore production which is an important virulence factor of *Pseudomonas* spp. [85]. In addition, honey inhibits bacterial adhesion to keratinocytes as well as the tissue proteins fibronectin, fibrinogen, and collagen, thus rendering wound pathogens less virulent [86]. Moreover, honey suppresses flagella-associated genes (*fleQ*, *fleN*, *fliA*, *fliC*) leading to the deflagellation of *Pseudomonas aeruginosa* and subsequently reduced motility, adherence, and virulence [87].

There is a 100-fold difference in antimicrobial potency between different honey types [88]. Manuka honey is probably the most often used for wound care because this was the first honey extensively investigated regarding the antimicrobial properties [24,89]. Furthermore, other types of honey have similar or superior antimicrobial activity and may be more effective for wound healing [22,90,91,92,93,94]. Current findings are in accordance with previous in vitro results that directly compared the antimicrobial activity of L-Mesitran Soft and Medihoney against eleven *Staphylococci* and eleven *Pseudomonas* spp. Pathogens [24]. L-Mesitran Soft was consistent in its activity and was more effective than Medihoney at roughly a two-fold stronger dilution, despite already containing half the concentration of honey [24]. Based on the differences in antimicrobial activity between the tested wound care products, we would suggest being careful in selecting the appropriate product. The main difference between Manuka and other types of MGH is their antimicrobial mechanism. Manuka-honey-mediated antimicrobial activity is mainly attributed to methylglyoxal whereas other honey types are mainly due to hydrogen peroxide [94,95,96].

Most studies demonstrate that regardless of the honey type, a concentration of at least 40% honey is sufficient to kill almost all tested microorganisms [97,98,99,100]. Therefore, a concentration above 40% will not further enhance the antimicrobial activity and is not needed in MGH formulations. In contrast, high concentrations of Manuka honey may be counterproductive and are associated with cytotoxicity, most likely as a result of the toxicity exerted by methylglyoxal, which is absent in other honey types [101,102,103]. It has been suggested to use at least 25% honey for therapeutic purposes [104] or at least 33% for being effective against biofilms [101]. The advantage of using a concentration of 40% honey is that other useful ingredients can be added, for example, to enhance the antimicrobial or wound-healing activity or to make it easier to apply. Pure honey is very sticky and sensitive to temperature influences; honey is hard when it is cold and drippy under warm conditions, affecting its applicability. Adding ingredients to apply it easier would benefit the wound care product [19]. Based on this, L-Mesitran Soft, in addition to its superior antimicrobial activity, will generally be more practical than pure honey formulations. Clinical efficacy is also supported by the presented cases.

## 4. Materials and Methods

### 4.1. Evaluation of Biofilm Inhibitory and Eradicating Activity of MGH-Containing Wound Care Products Using an In Vitro Wound Model

*Pseudomonas aeruginosa* PAO1 and *Staphylococcus aureus* Mu50 were grown aerobically on Tryptic Soy Agar at 37 °C. Biofilms were grown in an in vitro wound biofilm model containing an artificial dermis (AD) as described previously [31]. In brief, an overnight culture of the tested strain was pelleted, washed, resuspended in physiological saline (PS), and diluted to 10^6^ CFU per mL (as determined using the measurement of the optical density (OD) and a previously established calibration curve linking OD and number of CFU/mL). The ADs were soaked into a chronic wound biofilm medium (Bolton Broth with 50% plasma, 5% freeze–thaw laked horse blood, and 10 U/mL heparin). They were placed in the wells of a 24-well microtiter plate (TPP), and 10 μL aliquots of the cell suspension were spotted on each AD. A 0.5 mL volume of chronic wound biofilm medium was added to each well. The sterility of AD, wound biofilm medium, and wound care products were confirmed.

To evaluate the possible inhibitory effect of the products on the formation of the biofilm, 250 mg of product (see Table 1) was placed on each AD immediately after inoculation, and the biofilm model was incubated for 24 h at 37 °C to allow biofilm formation on the AD.

To evaluate the eradicating effects of the products (Table 1) on the biofilm, biofilms were first allowed to form for 24 h, after which the product was applied as described above and incubated for an additional 24 h.

In order to measure the inhibitory and eradicating activity after treatment, the entire content of the well was removed and placed in 10 mL of PS. Aggregates were dislodged by repeated vortexing and sonication (three cycles of 30 s vortexing and sonication; sonicator type: Branson Ultrasonic bath (Hach Company, Loveland, CO, USA)) and the number of CFU was determined by plating the resulting suspensions. The tested products were related to the untreated control, and the log reduction is presented in the results.

### 4.2. Honey Samples and Other Test Conditions

The antimicrobial activity of nine different products was assessed, including six wound care products and three different ingredient combinations used in L-Mesitran Soft and compared to the untreated control (Table 1).

### 4.3. Case Series

To illustrate the anti-biofilm activity of L-Mesitran Soft in the clinic, four patients that came to the wound care clinic and presented with wounds that presumably were infected with *Pseudomonas aeruginosa* biofilms were included. Pictures at the start of treatment and during follow up, showing a clear wound progression, will be presented. The patients were informed about the study, and they all gave written informed consent to use their photos and data for publication. The principles of the World Medical Association’s Declaration of Helsinki were followed.

### 4.4. Statistics

Statistical analysis was performed using GraphPad Prism 5.01 software (San Diego, CA, USA). Data were analyzed using one-way analysis of variance (ANOVA) with Bonferroni’s multiple comparison post hoc test as a correction for multiple comparisons. Results were considered significantly different at *p* < 0.05 (* *p* < 0.05, ** *p* < 0.01, and *** *p* < 0.001).

## 5. Conclusions

All tested MGH-based wound care products form a potent treatment for *Pseudomonas aeruginosa* biofilm inhibition and eradication. Nevertheless, there is a variability of antibiofilm activity between different wound care formulations depending on their compositions. Distinct ingredients, such as vitamins C and E, exert separate antimicrobial activity. However, in combination, a synergistic activity may occur. L-Mesitran Soft demonstrated the strongest activity because of its supplements. This efficacy has also been demonstrated in clinical cases of persistent biofilms without the need of using antibiotics.

## Figures and Tables

**Table 1:** Wound care products used in this study.

Products (Manufacturer)	Ingredients	Remark
Untreated control	Not applicable	No product
Medihoney (Derma Sciences, Inc., Toronto, Canada)	100% Manuka (*Leptospermum scoparium*) honey	Tube, Lot#: 1937 Expiry date: 2022-09
Revamil (BFactory Health Products B.V., Rhenen, the Netherlands)	100% pure medicinal honey	Tube, Lot#: AFR Expiry date: 2022-09
MEBO (Moist Exposed Burn Ointment) (Gulf Pharmaceutical Industries, Ras Al Khaimah, United Arab Emirates)	The base of the ointment is composed of beeswax and sesame oil. The main active ingredient is 0.25% beta-sitosterol. Other ingredients include eighteen amino acids, four major fatty acids, vitamins, trace elements, and polysaccharides.	Unknown
Melladerm Plus (SanoMed Manufacturing bv, Oostburg, the Netherlands)	45% honey, glycerine, propylene glycol, and PEG 4000	Tube, Lot#D8250311 Expiry date: 2023-04
L-Mesitran Ointment (Theo Manufacturing B.V., Maastricht, the Netherlands)	48% MGH, vitamins C and E, medical-grade hypoallergenic lanolin, sunflower oil, cod liver oil, Calendula officinalis, Aloe barbadensis, and zinc oxide	Tube, Lot#: 0020H Expiry date: 2021-12
L-Mesitran Soft (Theo Manufacturing B.V., Maastricht, the Netherlands)	40% MGH, vitamins C and E, medical-grade hypoallergenic lanolin, PEG 4000, and propylene glycol	Tube, Lot#: 201908X Expiry date: 2022-07
Raw honey	40% raw MGH, obtained from the same batch as L-Mesitran Ointment and L-Mesitran Soft (the concentration is similar as in L-Mesitran Soft)	Provided by Triticum Exploitatie BV
Vitamins C and E	Used at the same dose as present in L-Mesitran Soft	Provided by Triticum Exploitatie BV
Ingredients L-Mesitran Soft without the MGH	All ingredients at the same dose as used in L-Mesitran Soft, excluding MGH	Provided by Triticum Exploitatie BV

## Supplementary Materials

The following are available online at https://www.mdpi.com/2079-6382/9/12/866/s1, Figure S1: Antimicrobial activity of different natural products on *Staphylococcus aureus* (Mu50) biofilms.
