# Supplementary material for: Synergistic Antimicrobial Activity of Supplemented Medical-Grade Honey against Pseudomonas aeruginosa Biofilm Formation and Eradication
Source: Antibiotics (Basel). 2020 Dec 4;9(12):866. doi: 10.3390/antibiotics9120866 (PMC7761815; doi:10.3390/antibiotics9120866)
Supplement: Supplementary file 1 [file antibiotics-09-00866-s001.pdf]

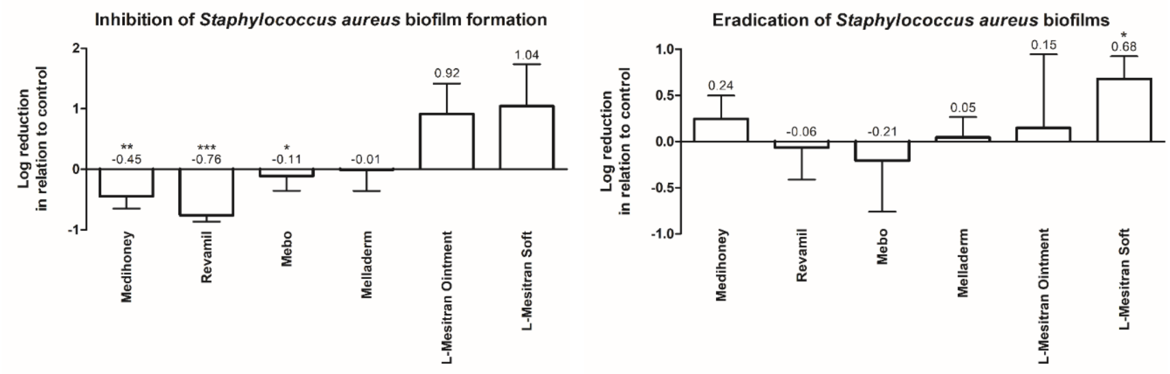

(**a**) (**b**)

Figure S1. Antimicrobial activity of different natural products on *Staphylococcus aureus* (Mu50) biofilms. Data are presented as mean log reduction ± standard deviation in relation to control. (**a**) Inhibition of *Staphylococcus aureus* biofilm formation. L-Mesitran Soft was significantly stronger compared to control, Medihoney, Revamil, and Mebo, as indicated by * above each column (*$p < 0.05$, **$p < 0.01$, ***$p < 0.001$). L-Mesitran Ointment was significantly stronger than Medihoney (*$p < 0.05$) and Revamil (*$p < 0.01$) (not shown in the graph). No other significant differences were observed. (**b**) Eradication of *Staphylococcus aureus* biofilms. L-Mesitran Soft was significantly stronger (*$p < 0.05$) compared to the control group as indicated by * above the column. No other significant differences were observed.
